# Identifying Predictive Factors for Complete Skin Clearance at Week 52 by Deucravacitinib in Moderate-to-Severe Psoriasis: A Prospective Observational Study

**DOI:** 10.7759/cureus.86044

**Published:** 2025-06-15

**Authors:** Yohei Takahashi, Teppei Hagino, Hidehisa Saeki, Eita Fujimoto, Naoko Kanda

**Affiliations:** 1 Dermatology, Nippon Medical School Chiba Hokusoh Hospital, Inzai, JPN; 2 Dermatology, Nippon Medical School, Tokyo, JPN; 3 Dermatology, Fujimoto Dermatology Clinic, Funabashi, JPN

**Keywords:** complete skin clearance, deucravacitinib, predictive factor, psoriasis, tyrosine kinase 2

## Abstract

Background: Deucravacitinib, an oral selective tyrosine kinase 2 inhibitor, is effective for moderate-to-severe psoriasis. However, not all patients can achieve complete skin clearance (CSC) by deucravacitinib even after long-term treatment (one year). Therefore, we sought to determine predictive baseline factors of achieving CSC (Psoriasis Area and Severity Index score 0) at week 52.

Methods: In this prospective single‑center study (December 2022 - February 2025), we enrolled 77 patients aged ≥ 15 years and treated them with deucravacitinib 6 mg once daily. Baseline demographic, clinical, and laboratory factors were compared between patients who achieved CSC at week 52 and those who did not.

Results: Seventy-seven patients aged ≥ 15 years with moderate to severe psoriasis received deucravacitinib 6 mg once daily, and 15 patients achieved CSC at week 52. We compared patients’ background factors and baseline clinical and laboratory indices between week 52 CSC achievers and non-achievers. The age (years) of week 52 CSC achievers (median: 55.0; IQR: 45.0-65.5) was significantly younger compared to non-achievers (median: 67.0; IQR: 55.3-76.0) (p = 0.0461, by Mann-Whitney U test; area under the curve (AUC) = 0.667). Additionally, the body mass index (BMI) of week 52 CSC achievers (median: 20.9; IQR: 18.3-22.9) was significantly lower compared to non-achievers (median: 23.6; IQR: 22.0-27.1) (p = 0.00571, by Mann-Whitney U test; AUC = 0.733). Compared to non-achievers, week 52 CSC achievers showed higher and faster improvements of nail, scalp, and genital lesions and quality of life, evaluated by the Dermatology Life Quality Index.

Conclusions: A BMI < 22.9 kg/m² and age < 61 years independently identify patients most likely to obtain CSC at week 52 with deucravacitinib. These cut‑offs can support early treatment selection and stratification in future prospective trials, facilitating personalized care for psoriasis.

## Introduction

Psoriasis is a chronic inflammatory skin disease characterized by abnormal activation of the inflammatory interleukin (IL)-23/IL-17 axis [1-4]. The prevalence of psoriasis is high worldwide, i.e., 2-4% in Western countries and 0.3-0.4% in Japan [5-7]. Patients with moderate to severe plaque psoriasis often experience impaired quality of life (QoL) due to troublesome symptoms and feelings of stigmatization or depression. For these patients, biologics targeting IL-23, IL-17, or tumor necrosis factor (TNF)-α have improved symptoms and QoL. Especially, achieving complete skin clearance (CSC) (Psoriasis Area and Severity Index (PASI) score = 0, which indicates that no visible psoriatic lesions remain) by biologics resulted in reduced negative emotions and increased confidence of patients, with the greatest benefits to QoL [8,9]. Thus, achievement of CSC is recommended as a treatment goal of biologic therapies for psoriasis. However, many patients still evade injections, and anticipate oral medicine as effective and safe as biologics.

Deucravacitinib is an oral agent approved for moderate to severe psoriasis and selectively inhibits tyrosine kinase 2 (TYK2) [10]. TYK2 belongs to the Janus kinase (JAK) family and is involved in signaling pathways of cytokines, IL-12, IL-23, and type I interferons (IFNs), which play key roles in the pathogenesis of psoriasis [11]. Unlike conventional JAK1/2/3 inhibitors that bind the highly conserved adenosine triphosphate (ATP)‑binding catalytic domain and may therefore inhibit multiple JAKs, deucravacitinib binds the less‑conserved regulatory (pseudokinase) domain of TYK2, resulting in high selectivity for TYK2 and a lower risk of off‑target JAK‑related adverse events. Phase 3 clinical trials in patients with moderate-to-severe plaque psoriasis demonstrated high efficacy and safety of deucravacitinib treatment [12-16]. In real-world settings, this treatment has been reported to be well tolerated and generate favorable outcomes reported by both clinicians and patients [17]. Notably, deucravacitinib improved symptoms of scalp, nails, and genital regions and QoL at weeks 24 and 52 of treatment [18-22]. These findings suggest that deucravacitinib could be a useful oral treatment for psoriasis, comparable to biologics. Systemic inflammatory indices such as the systemic immuno-inflammatory index (SII) and systemic inflammatory response index (SIRI) are known to reflect whole-body inflammation and have been used as severity markers in inflammatory diseases, as well as prognostic markers in cancer and cardiovascular disease [23].

On the other hand, the responsiveness to deucravacitinib varies among patients with psoriasis, and not all patients can achieve CSC even after its long-term (around one year) treatment in real-world settings. It is thus useful to clarify the patients’ baseline features that predict the achievement of CSC by long-term treatment with deucravacitinib. To our knowledge, no prospective observational study has yet examined baseline predictors of achieving PASI 0 with deucravacitinib after 52 weeks of treatment. Such predictive factors may help optimize patient selection for this treatment.

The aim of this study is to determine predictive baseline factors for the achievement of CSC (PASI = 0) at week 52 of deucravacitinib treatment.

## Materials and methods

Study design

A prospective single-center study was conducted in Japan from December 2022 to February 2025. This study included patients aged ≥ 15 years diagnosed with moderate or severe psoriasis, based on clinical findings. After a comprehensive discussion with clinicians, patients themselves chose deucravacitinib among various systemic therapies. All patients received 6 mg of oral deucravacitinib once daily, without topical agents.

Data collection

At week 0, demographic and clinical characteristics such as age, sex, body mass index (BMI), duration of psoriasis, and presence of arthritis, scalp, nail, or genital lesions were recorded. Comorbidities, including diabetes or cardiovascular diseases, smoking status, and prior use of systemic therapy (apremilast or biologics), were also documented. Clinical indices included PASI and Dermatology Life Quality Index (DLQI). PASI was scored at every visit by the treating dermatologist, and independent, blinded raters were not employed. Laboratory indices included lactate dehydrogenase (LDH), C-reactive protein (CRP), neutrophil-to-lymphocyte ratio, monocyte-to-lymphocyte ratio, platelet-to-lymphocyte ratio, SII (calculated as follows: neutrophil count × platelet count/lymphocyte count), and SIRI (calculated as follows: neutrophil count × monocyte count/lymphocyte count). Blood samples were collected in the fasting state and processed according to standard laboratory procedures. These clinical and laboratory indices were re-evaluated at weeks four, 16, 28, 40, and 52 to track treatment response over time.

Inclusion criteria

Patients clinically diagnosed with plaque psoriasis, psoriatic arthritis, or psoriatic erythroderma were included. Psoriatic arthritis was diagnosed using the classification criteria for psoriatic arthritis. Psoriatic erythroderma was defined as diffuse erythema and scaling involving > 90% of the body surface area (BSA), where BSA denotes the percentage of total skin surface affected. All patients in this study received deucravacitinib for at least 52 weeks. Since this was not a clinical trial, there was no strict washout period when switching from other systemic therapies (apremilast or biologics) to deucravacitinib. Therefore, clinical indices, PASI and DLQI, before switching to deucravacitinib were not tracked, and the time of switching to deucravacitinib was defined as baseline (week 0).

Exclusion criteria

Patients who discontinued and resumed deucravacitinib were excluded. Other exclusion criteria were malignancy, serious cardiovascular diseases (heart failure, myocardial infarction, or stroke), active infections, including tuberculosis, previous hypersensitivity to deucravacitinib or its components, and pregnancy or breastfeeding.

Definition of long-term CSC achievers

We defined long-term CSC achievers as patients who achieved a total (whole body) PASI score of 0 at week 52. We compared baseline clinical or laboratory indices and background factors between long-term CSC achievers and non-achievers (patients who did not achieve a PASI score of 0 at week 52).

We also evaluated total and site-specific PASI scores (head and neck, trunk, upper limbs, and lower limbs) and DLQI scores at weeks 0, 4, 16, 24, 36, and 52, and compared these scores between long-term CSC achievers and non-achievers. In patients having scalp, nail, or genital lesions, their severity was assessed by five-point scales (0 to 4). We calculated achievement rates of scalp-specific physician’s global assessment (PGA) = 0 (ss-PGA 0), PGA of fingernail psoriasis = 0 (PGA-F 0), and genital PGA = 0 (genital PGA 0), and compared the rates between long-term CSC achievers and non-achievers.

Ethical considerations

This study was conducted in accordance with the Declaration of Helsinki (2004) and was approved by the institutional review board of the participating facility (Nippon Medical School Chiba Hokusoh Hospital; Approval No.: H-2023-095). Written informed consent was obtained from all participants in advance.

Statistical analysis

Results are expressed as mean ± standard deviation for variables with a normal distribution, and as median and interquartile range (IQR) for variables with a nonparametric distribution. Differences between the two groups were assessed using Student’s t-test for variables with a normal distribution, and the Mann-Whitney U test for variables with a non-parametric distribution. Fisher’s exact test was used to evaluate differences in the frequencies of categorical variables. A p-value < 0.05 was considered statistically significant. Receiver operating characteristic (ROC) analysis was performed to evaluate the predictive ability of each variable for CSC achievement at week 52 by deucravacitinib. ROC curves were generated only for variables that reached significance in the univariate tests (age and BMI). The area under the curve (AUC) was used to determine predictive ability. All statistical analyses were performed using Easy R (Jichi Medical University Saitama Medical Center, Saitama, Japan).

## Results

Baseline characteristics

This study included 77 patients with psoriasis. Among them, 58/77 (75.3%) were male (Table 1). The median age of the patients was 62.0 (IQR: 51.0-76.0) years, the median BMI was 23.0 (IQR: 20.1-25.8) kg/m^2^, and the median disease duration was 7.0 (IQR: 2.5-23.0) years. Most patients (53/77, 68.8%) had plaque psoriasis, 20/77 (26.0%) had psoriatic arthritis, and 4/77 (5.2%) had erythrodermic psoriasis. Prior treatments included topical corticosteroid and/or vitamin D3 alone (43/77, 55.8%), apremilast (22/77, 28.6%), adalimumab (1/77, 1.3%), bimekizumab (4/77, 5.2%), ixekizumab (2/77, 2.6%), guselkumab (3/77, 3.9%), and tildrakizumab (5/77, 6.5%). The baseline PASI score was 14.4 ± 9.5, and the baseline DLQI score was 8.7 ± 5.5. Scalp, nail, and genital lesions were seen in 70/77 patients (90.9%), 45/77 patients (58.4%), and 47/77 patients (61%), respectively.

**Table 1:** Baseline characteristics of total patients. Data are presented as n (%) unless otherwise indicated. BMI, body mass index; DLQI, Dermatology Life Quality Index; IQR, interquartile range; SD, standard deviation; PASI, Psoriasis Area and Severity Index; NLR, neutrophil-to-lymphocyte ratio; MLR, monocyte-to-lymphocyte ratio; PLR, platelet-to-lymphocyte ratio; SII, systemic immuno-inflammatory index, calculated as neutrophil count × platelet count/lymphocyte count; SIRI, systemic inflammatory response index, calculated as neutrophil count × monocyte count/lymphocyte count.

Characteristic	Total patients (n = 77)
Male sex	58 (75.3%)
Age (years), median (IQR)	62.0 (51.0 – 76.0)
BMI (kg/m^2^),median (IQR)	23.0 (20.1 – 25.8)
Disease duration (years),median (IQR)	7.0 (2.5 – 23.0)
Type of psoriasis	
Plaque psoriasis	53 (68.8)
Psoriatic arthritis	20 (26.0)
Erythrodermic psoriasis	4 (5.2)
Presence of difficult-to-treat lesions	
Scalp lesions	70 (90.9)
Nail lesions	45 (58.4)
Genital lesions	47 (61.0)
Other characteristics	
Current smokers	37 (48.1)
Diabetes mellitus	5 (6.5)
Cardiovascular diseases	9 (11.7)
Prior treatments	
Topical corticosteroid and/or vitamin D3 alone	43 (55.8)
Apremilast	22 (28.6)
Adalimumab	1 (1.3)
Bimekizumab	4 (5.2)
Ixekizumab	2 (2.6)
Guselkumab	3 (3.9)
Tildrakizumab	5 (6.5)
Baseline clinical indices	
DLQI, mean ± SD	8.7 ± 5.5
PASI, mean ± SD	14.4 ± 9.5
Baseline laboratory indices	
Lactate dehydrogenase (IU/mL), median (IQR)	183.0 (162.0 – 215.0)
C-reactive protein (mg/dL), median (IQR)	0.1 (0.1 – 0.4)
NLR, median (IQR)	2.5 (2.0 – 3.8)
MLR, median (IQR)	0.3 (0.2 – 0.5)
PLR, median (IQR)	156.5 (120.2 – 246.4)
SII, median (IQR)	569.1 (424.9 – 1017.5)
SIRI, median (IQR)	1.1 (0.8 – 1.8)

Comparison of baseline features between long-term CSC achievers versus non-achievers

Among 77 patients, 15 patients achieved CSC at week 52 while 62 patients did not (Table 2). Age of long-term CSC achievers (median: 55.0; IQR: 45.0-65.5 years) was significantly younger compared to non-achievers (median: 67.0; IQR: 55.3-76.0 years) (p = 0.0461, by Mann-Whitney U test). Additionally, the BMI of long-term CSC achievers (median: 20.9; IQR: 18.3-22.9 kg/m^2^) was significantly lower compared to non-achievers (median: 23.6; IQR: 22.0-27.1 kg/m^2^) (p < 0.01, by Mann-Whitney U test). No significant differences were observed in the other baseline features.

**Table 2:** Comparison of baseline features between achievers of complete skin clearance (CSC) at week 52 versus non-achievers by deucravacitinib treatment for psoriasis. ^a^ Data provided as median (interquartile range). * p < 0.05 and ** p < 0.01 by Mann-Whitney U test or Fisher’s exact test. Female sex: CSC achievers 5/15 (33.3%) and non‑achievers 14/62 (22.6%). PASI, Psoriasis Area and Severity Index; DLQI, Dermatology Life Quality Index; NLR, neutrophil-to-lymphocyte ratio; MLR, monocyte-to-lymphocyte ratio; PLR, platelet-to-lymphocyte ratio; SII, systemic immuno-inflammatory index, calculated as neutrophil count × platelet count/lymphocyte count; SIRI, systemic inflammatory response index, calculated as neutrophil count × monocyte count/lymphocyte count.

Background factors or baseline values of clinical or laboratory indices	CSC achievers (n = 15)	Non-achievers (n = 62)	p
Male sex, n (%)	10 (66.7)	48 (77.4)	0.505
Presence of scalp lesions, n (%)	13 (86.7)	57 (91.9)	0.617
Presence of nail lesions, n (%)	7 (46.7)	38 (61.3)	0.385
Presence of genital lesions, n (%)	9 (60.0)	38 (61.3)	1
Presence of arthritis, n (%)	2 (13.3)	18 (29.0)	0.328
Current smoking, n (%)	6 (40.0)	31 (50.0)	0.572
Diabetes mellitus, n (%)	0	5 (8.1)	0.576
Cardiovascular disease, n (%)	0	9 (14.5)	0.192
Previous apremilast treatment, n (%)	4 (26.7)	18 (29.0)	1
Previous biologic treatment, n (%)	2 (13.3)	12 (19.4)	0.725
Age (years) ^a^	55.0 (45.0 – 65.5)	67.0 (55.3 – 76.0)	0.0461*
Body mass index (kg/m^2^) ^a^	20.9 (18.3 – 22.9)	23.6 (22.0 – 27.1)	0.00571**
Disease duration (years) ^a^	6.0 (1.5 – 20.0)	7.0 (3.0 – 30.0)	0.481
PASI ^a^	11.5 (5.1 – 15.5)	13.3 (7.3 – 22.3)	0.297
DLQI ^a^	9.0 (4.5 – 10.0)	7.0 (5.0 – 10.0)	0.684
Lactate dehydrogenase (IU/mL)^ a^	177.0 (160.0 – 204.5)	188.0 (162.0 – 213.8)	0.457
C-reactive protein (mg/dL)^a^	0.2 (0.2 – 0.4)	0.1 (0.1 – 0.3)	0.994
NLR ^a^	2.3 (1.8 – 2.7)	2.6 (2.0 – 3.9)	0.159
MLR^ a^	0.2 (0.2 – 0.4)	0.3 (0.3 – 0.5)	0.0713
PLR ^a^	156.5 (120.2 – 182.8)	158.1 (126.4 – 257.1)	0.642
SII ^a^	489.7 (422.0 – 730.0)	598.8 (425.7 – 1019.8)	0.592
SIRI ^a^	0.8 (0.6 – 1.3)	1.3 (0.9 – 1.8)	0.118

ROC curve analysis for predicting long-term CSC achievers

**Figure 1:**
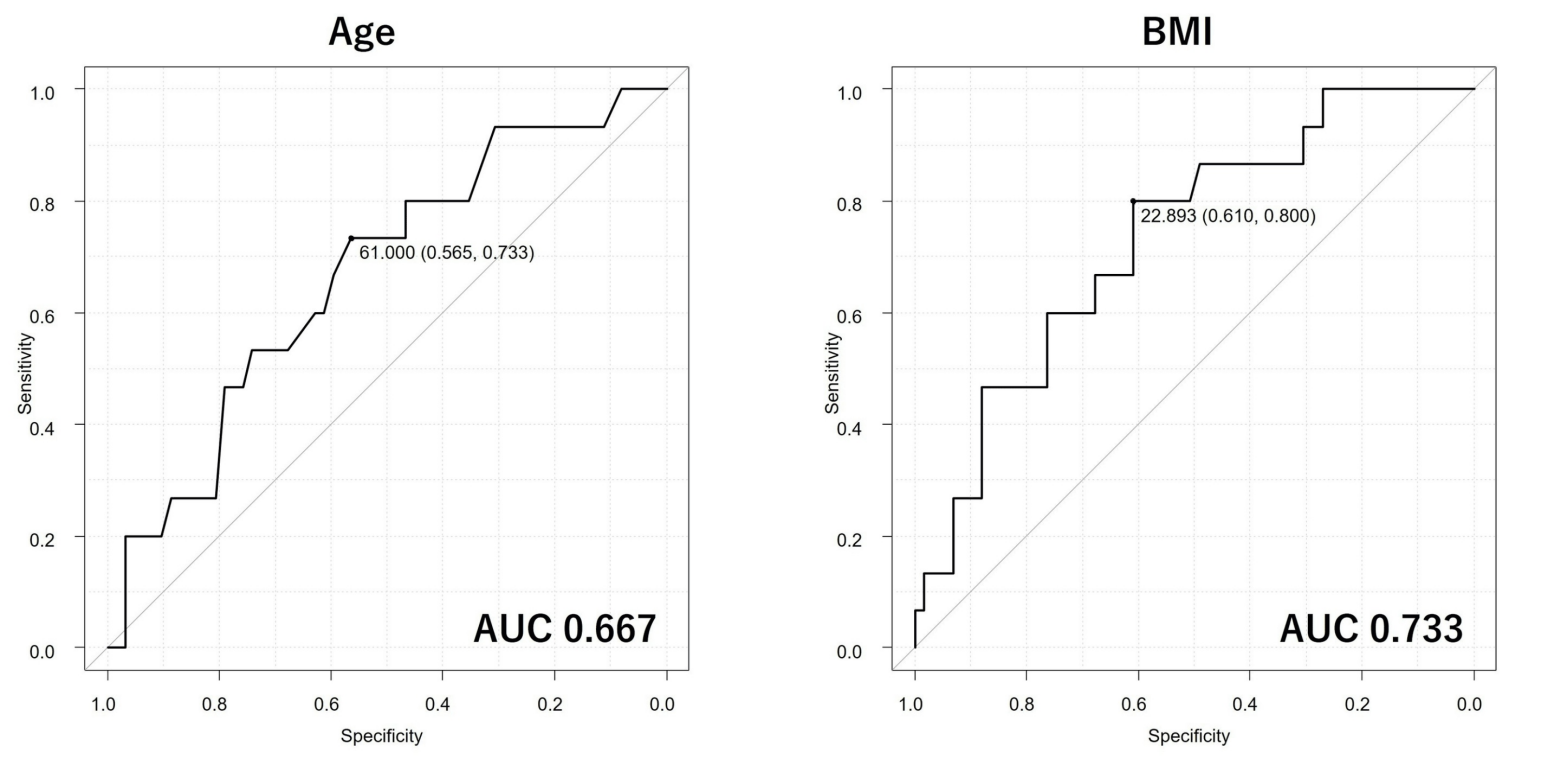
The receiver operating characteristic curves for evaluating the predictive abilities of age and body mass index (BMI) for complete skin clearance at week 52 of deucravacitinib treatment. The area under the curve (AUC) for age is 0.667 (95% CI: 0.516–0.819), and that for BMI is 0.733 (95% CI: 0.596–0.870).

ROC curve analysis was performed to assess the predictive abilities of age and BMI in distinguishing long-term CSC achievers from non-achievers (Figure 1). The AUC for age was 0.667 (95% confidence interval (CI): 0.516-0.819), with specificity of 73.3% and sensitivity of 56.5% by a cut-off value of 61.0 years. The AUC for BMI was 0.733 (95% CI: 0.596-0.87), with specificity of 80.0% and sensitivity of 61.0% by a cut-off value of 22.893 kg/m^2^. Based on AUCs, the predictive ability of age was low while that of BMI was moderate.

Transition of PASI and DLQI scores in long-term CSC achievers compared to non-achievers

**Figure 2:**
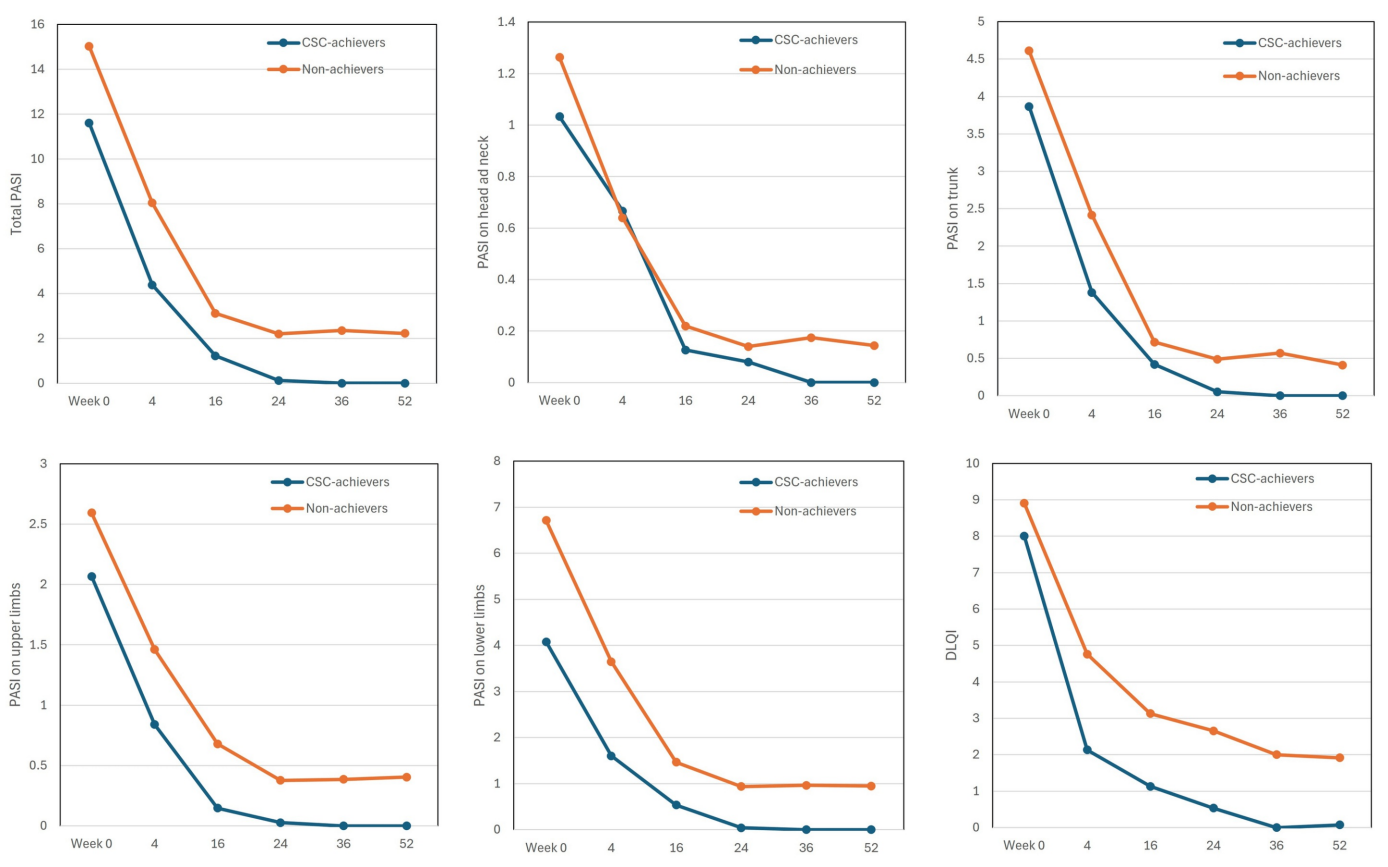
The transitions of total Psoriasis Area and Severity Index (PASI), PASI on individual anatomical sites (head and neck, trunk, upper limbs, lower limbs), and Dermatology Life Quality Index (DLQI) in long-term complete skin clearance (CSC) achievers (n = 15, blue) and non-achievers (n = 62, orange) during the treatment with deucravacitinib 6 mg. Data are mean values at weeks 0, 4, 16, 24, 36, and 52.

Total PASI scores and PASI scores of all four anatomical sites decreased steadily in both long-term CSC achievers and non-achievers from week four onward (Figure 2). However, at week 24, the decrease of total and site-specific PASI scores in non-achievers was stopped, while in all long-term CSC achievers, PASI scores of trunk and upper and lower limbs declined to 0 at week 24, and total and head/neck PASI scores declined to 0 at week 36.

DLQI scores rapidly decreased at week four and continued to decrease through week 52 in both long-term CSC achievers and non-achievers. However, the mean DLQI in long-term CSC achievers declined to 0 at week 36, while that in non-achievers remained around 2 even at week 52. DLQI 0 corresponds to the experience of no impairment on QoL. The achievement rate of DLQI 0 at week 52 in long-term CSC achievers (13/14, 92.9%) was significantly higher than that in non-achievers (7/59, 11.9%) (p < 0.01, by Fisher’s exact test). These findings indicate that long-term CSC achievers obtained more pronounced improvement of rash and QoL compared to non-achievers throughout 52 weeks of deucravacitinib treatment.

Achievement rates of ss-PGA 0, PGA-F 0, and genital PGA 0 in long-term CSC achievers compared to non-achievers

**Figure 3:**
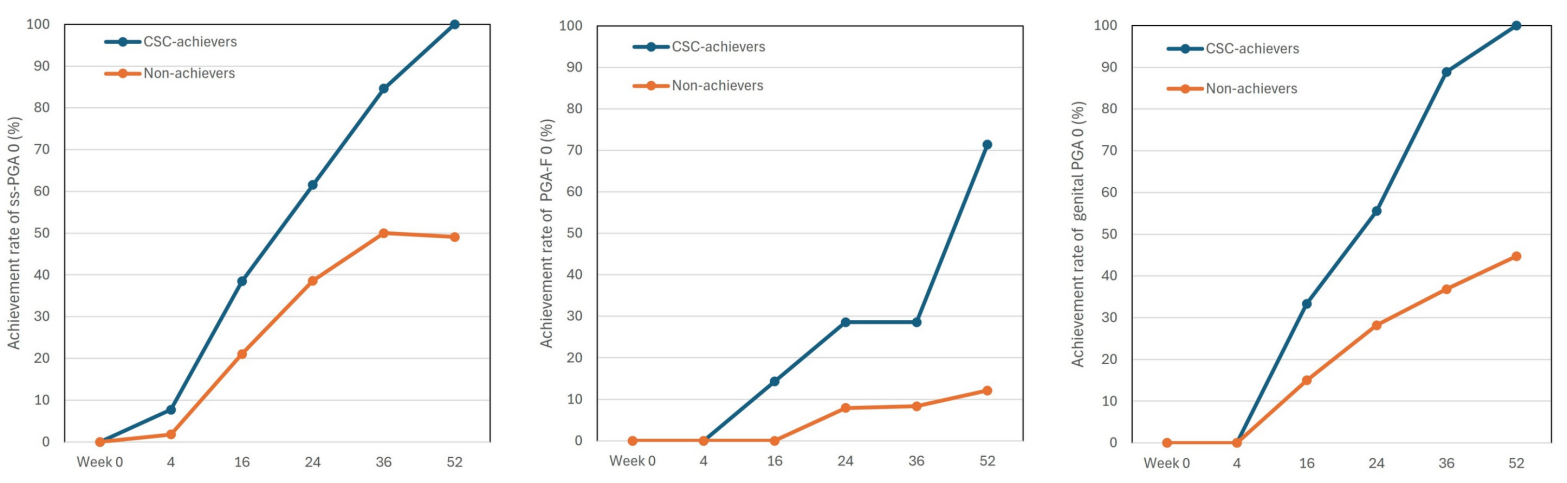
The achievement rates of scalp-specific physician’s global assessment (ss-PGA) 0, PGA of fingernail psoriasis (PGA-F) 0, and genital PGA 0 during the treatment with deucravacitinib 6 mg. Data are percentages for long-term CSC achievers (blue) and non-achievers (orange) at weeks 0, 4, 16, 24, 36, and 52. The number of long-term CSC achievers and non-achievers was 13 and 57 in scalp analysis, seven and 38 in fingernail analysis, and nine and 38 in genital analysis, respectively. CSC, complete skin clearance; PGA, physician’s global assessment.

From week 16 onward, achievement rates of ss-PGA 0, PGA-F 0, and genital PGA 0 rapidly increased in long-term CSC achievers, while non-achievers showed much slower increases. The differences in the achievement rates between the two populations widened further from week 16 onwards. The speed for achieving PGA-F 0 appeared slower compared to ss-PGA 0 and genital PGA 0. At week 52, the achievement rates of ss-PGA 0, PGA-F 0, and genital PGA 0 in long-term CSC achievers (9/9 (100%), 5/6 (83.3%), and 9/9 (100%), respectively), were much higher than those in non-achievers (26/53 (49.1%), 4/30 (13.3%), and 17/38 (44.7%), respectively)　and these differences were statistically significant (p < 0.01, by Fisher’s exact test). These findings suggest that long-term CSC achievers showed faster and more substantial improvement in scalp, nail, and genital lesions compared to non-achievers throughout 52 weeks of deucravacitinib treatment (Figure 3).

## Discussion

In this study, achievers of CSC (PASI = 0) at week 52 of deucravacitinib treatment showed lower BMI compared to non-achievers. Prior studies also reported that achievers of investigator’s global assessment (IGA) 0/1 had lower BMI compared to non-achievers at week 16 of deucravacitinib treatment [24]. In addition, a real-world long-term analysis revealed that psoriasis patients with a BMI < 25 kg/m^2^ had higher achievement rates of PASI 100 at week 52 (29.41%) than those with a BMI ≥ 25 kg/m^2^ (18.18%) [25]. Increase of BMI is associated with hypertrophy of visceral adipose tissues overproducing inflammatory cytokines, IL-23, IL-12, TNF-α, IL-18 [26] or IFN-α [27]. Deucravacitinib suppresses the downstream signaling of TYK2-dependent cytokines, IL-23, IL-12, or type I IFNs. Thus, patients with lower BMI may have lower levels or activities of the above cytokines, and thus be more susceptible to the inhibitory effects of deucravacitinib. Further, patients with lower BMI may obtain higher per kg dose of orally taken deucravacitinib, and may generate higher concentrations of deucravacitinib at lesional skin, which may also contribute to the higher effectiveness. In this study, the BMI cut-off for week 52 CSC was 22.89 kg/m^2^; however, the predictive accuracy was moderate (AUC: 0.733). We recommend conducting larger, multicenter cohort studies to refine the cut-off value for predicting long-term CSC achievement with deucravacitinib.

Week 52 CSC achievers by deucravacitinib had a younger age compared to non-achievers. In contrast, a previous real-world study reported that achievers of IGA 0/1 had an older age compared to non-achievers at week 16 of deucravacitinib treatment [24]. This discrepancy indicates that a younger age might not be favorable for early partial clearance of rash, such as IGA = 1 (almost clear skin), but could be essential for achieving IGA = 0 (CSC) by a long-term treatment with deucravacitinib. In treat-to-target management of psoriasis, IGA 0/1 corresponds to PASI ≤ 2, which almost aligns with PASI 90 [28]. Thus, patients’ population achieving near-CSC (PASI = 1 or 2) at earlier timepoints may differ in age from that achieving CSC (PASI = 0) at week 52 of deucravacitinib treatment. Similar observations have been reported in the GUIDE trial of anti-IL-23 antibody guselkumab; each year of increasing age (odds ratio (OR) = 0.98; 95% CI: 0.97-0.99; p < 0.001, by a multiple logistic regression) and each kg/m^2^ increase in BMI (OR = 0.95; 95% CI: 0.93-0.98; p < 0.001) decreased the odds of being a super responder (PASI = 0) at week 20 or 28, indicating the association of younger age and lower BMI with super-response (PASI = 0) to guselkumab [29]. Since deucravacitinib can inhibit the action of IL-23 by blocking TYK2, the CSC (PASI = 0) achievers by deucravacitinib may have the same characteristics as those of super-responders to guselkumab. Therefore, our results propose that younger patients may have a greater chance of week 52 CSC by deucravacitinib, even though older patients might attain near-CSC earlier. It is reported that healthy elderly people (≥65 years) showed increases of T helper 17 (Th17) frequency, RORγt expression and IL-17 level in peripheral blood compared to healthy middle-aged (45 to 64 years) and young people (≤44 years) [30], indicating lower Th17 activities in younger subjects. Thus, younger patients with psoriasis may be more susceptible to the inhibition of Th17 responses by IL-23 inhibitors, including deucravacitinib, and thus more liable to obtain CSC. The cut-off value of age for week 52 CSC by deucravacitinib was 61.0 years; however, the predictive ability was low based on AUC (0.667). We should further establish a more correct cut-off value by a larger cohort study.

Clinicians may apply the provisional thresholds (BMI < 22.9 kg/m² and age < 61 years) to set expectations: patients below both cut‑offs can be encouraged to persist with deucravacitinib, while those above either cut‑off should be counseled on a lower chance of complete clearance and monitored for early therapy adjustment if response is poor.

Although not statistically significant, baseline systemic‑inflammation markers (NLR, MLR, PLR, SII, and SIRI) tended to be lower in CSC achievers, suggesting that a generally milder inflammatory milieu may favor complete response and warrants confirmation in larger cohorts.

In recent years, treatment effectiveness has been linked to disease duration; however, we detected no significant difference between CSC achievers (median: 6.0 (1.5-20.0) years) and non‑achievers (median: 7.0 (3.0-30.0) years) (p = 0.481). The overlapping IQRs and limited sample size likely reduced statistical power.

Moreover, although not statistically significant, the median baseline PASI was lower in CSC achievers (11.5 (5.1-15.5)) than in non-achievers (13.3 (7.3-22.3)); this milder initial severity could partly explain their better long-term response and should be explored further in a larger cohort.

In this study, the decrease of DLQI paralleled that of PASI through 52 weeks of deucravacitinib treatment. Further, 92.9% of long-term CSC achievers obtained a DLQI score of 0 at week 52, while only 11.9% of non-achievers did. The present results on deucravacitinib are similar to those on biologics; higher achievement rate of DLQI 0 in patients with CSC (PASI 100) (54.4%) compared to those with near-CSC (PASI improvement = 90-99%) (37.7%) at week 12 of ixekizumab treatment [8] and higher rate of DLQI 0/1 in patients with PASI 100 (93%) compared to patients with PASI improvement = 90% (83.8%) at week 16 of bimekizumab treatment [9]. Our present results indicate that achieving CSC generates the greatest improvement of QoL and thus represents a clinically meaningful treatment goal for deucravacitinib treatment, similarly to biologics.

In this study, the achievement rates of ss-PGA 0, PGA-F 0, and genital PGA 0 in the long-term CSC achievers increased faster compared to non-achievers, and the rates at week 52 in the former were much higher than those in the latter. These findings suggest that CSC aligns with the clearance of difficult-to-treat lesions, scalp, nail, and genital lesions under deucravacitinib treatment, which may also contribute to the complete improvement of QoL since these lesions generate disfigurement or impaired sexual life, and extremely impact patients’ QoL.

This study has several limitations. First, we only included Japanese patients. We recommend multicenter studies with larger cohorts and genetic profiling to validate these findings. Second, it was conducted at a single center with a small sample size. No formal a priori sample size calculation was performed; the 77 patients represent all eligible cases during the enrolment period and provide exploratory, not definitive, power to detect group differences. Third, we used a standard method to measure CRP, although high-sensitivity CRP might be preferred. Fourth, the 52-week treatment period might be rather short to assess long-term outcomes. Fifth, we did not examine potential genetic factors. Sixth, the number of patients having scalp, nail, or genital lesions in long-term CSC achievers was small, which might influence the achievement rates of their clearance. Seventh, we were unable to quantify treatment response in facial lesions because few patients had clearly demarcated facial involvement; as a result, objective facial effectiveness data are lacking, and future studies focusing specifically on facial psoriasis are warranted. Eighth, our ROC analyses for age and BMI produced AUCs of 0.667 and 0.733, respectively, values that denote only modest predictive power; therefore, the proposed cut‑offs should be viewed as exploratory and require validation in larger, independent cohorts. Ninth, when we explored stratifying the cohort into two age groups (e.g., < 60 years and ≥ 60 years), the number of CSC achievers in each stratum was too small to permit a statistically reliable age‑adjusted BMI analysis. Larger multicenter studies are necessary to clarify the interaction between age and BMI. Tenth, we did not include an active comparator group (e.g., patients receiving biologics) or use a historical control cohort, limiting our ability to contextualize the effectiveness of deucravacitinib relative to other systemic therapies. Eleventh, 28.6% of participants had prior apremilast exposure, suggesting that our study cohort may differ from the general psoriasis population and potentially introducing selection bias that limits the generalizability of our findings. Twelfth, because treatment selection was driven by shared decision-making rather than random assignment, we recognize a potential selection bias: patients who chose deucravacitinib may differ in disease severity, prior treatment response, or preference compared with those who continued other systemic therapies. Thirteenth, odds ratios with confidence intervals were not available from the analytical software used (Easy R); further research using more advanced statistical tools is warranted to obtain robust effect‑size estimates. Finally, there was no strict washout period when switching from other systemic therapies to deucravacitinib.

## Conclusions

Based on this exploratory, single‑center cohort, younger age and lower BMI were the only baseline characteristics associated with CSC (PASI = 0) at week 52 of deucravacitinib treatment. CSC achievers also experienced numerically faster improvement in QoL and in scalp, nail, and genital lesions, but these secondary findings should be interpreted with caution, given the limited sample size and lack of a control group. Thus, the provisional age (<61 years) and BMI (<22.9 kg/m²) cut‑offs are hypothesis‑generating and require confirmation in larger, multi‑center studies before they can guide routine clinical decision‑making.
